# Whole blood microarray analysis of pigs showing extreme phenotypes after a porcine reproductive and respiratory syndrome virus infection

**DOI:** 10.1186/s12864-015-1741-8

**Published:** 2015-07-10

**Authors:** Martine Schroyen, Juan P. Steibel, James E. Koltes, Igseo Choi, Nancy E. Raney, Christopher Eisley, Eric Fritz-Waters, James M. Reecy, Jack C. M. Dekkers, Robert R. R. Rowland, Joan K. Lunney, Catherine W. Ernst, Christopher K. Tuggle

**Affiliations:** Department of Animal Science, Iowa State University, Ames, IA USA; Department of Animal Science, Michigan State University, East Lansing, MI USA; Department of Fisheries and Wildlife, Michigan State University, East Lansing, MI USA; APDL, BARC, ARS, USDA, Beltsville, MD USA; Department of Statistics, Iowa State University, Ames, IA USA; Department of Diagnostic Medicine/Pathobiology, College of Veterinary Medicine, Kansas State University, Manhattan, KS USA

**Keywords:** Pig, PRRS, Microarray, Transcriptomics, WGCNA, PCIT, Immune response

## Abstract

**Background:**

The presence of variability in the response of pigs to Porcine Reproductive and Respiratory Syndrome virus (PRRSv) infection, and recent demonstration of significant genetic control of such responses, leads us to believe that selection towards more disease resistant pigs could be a valid strategy to reduce its economic impact on the swine industry. To find underlying molecular differences in PRRS susceptible versus more resistant pigs, 100 animals with extremely different growth rates and viremia levels after PRRSv infection were selected from a total of 600 infected pigs. A microarray experiment was conducted on whole blood RNA samples taken at 0, 4 and 7 days post infection (dpi) from these pigs. From these data, we examined associations of gene expression with weight gain and viral load phenotypes. The single nucleotide polymorphism (SNP) marker WUR10000125 (WUR) on the porcine 60 K SNP chip was shown to be associated with viral load and weight gain after PRRSv infection, and so the effect of the WUR10000125 (WUR) genotype on expression in whole blood was also examined.

**Results:**

Limited information was obtained through linear modeling of blood gene differential expression (DE) that contrasted pigs with extreme phenotypes, for growth or viral load or between animals with different WUR genotype. However, using network-based approaches, molecular pathway differences between extreme phenotypic classes could be identified. Several gene clusters of interest were found when Weighted Gene Co-expression Network Analysis (WGCNA) was applied to 4dpi contrasted with 0dpi data. The expression pattern of one such cluster of genes correlated with weight gain and WUR genotype, contained numerous immune response genes such as cytokines, chemokines, interferon type I stimulated genes, apoptotic genes and genes regulating complement activation. In addition, Partial Correlation and Information Theory (PCIT) identified differentially hubbed (DH) genes between the phenotypically divergent groups. GO enrichment revealed that the target genes of these DH genes are enriched in adaptive immune pathways.

**Conclusion:**

There are molecular differences in blood RNA patterns between pigs with extreme phenotypes or with a different WUR genotype in early responses to PRRSv infection, though they can be quite subtle and more difficult to discover with conventional DE expression analyses. Co-expression analyses such as WGCNA and PCIT can be used to reveal network differences between such extreme response groups.

**Electronic supplementary material:**

The online version of this article (doi:10.1186/s12864-015-1741-8) contains supplementary material, which is available to authorized users.

## Background

In the United States, Porcine Reproductive and Respiratory Syndrome (PRRS) is one of the most economically devastating diseases currently in the swine industry [1, 2]. PRRS affects all production stages, manifesting reproductive losses (infertility, abortions, and stillborn and mummified fetuses), and piglets show a higher pre-weaning morbidity and mortality rate, persisting with a reduced thrift throughout the entire grow-finishing period [3]. The disease is caused by the PRRS virus (PRRSv), an enveloped, single stranded RNA virus that belongs to the Arteriviridae family [4, 5]. PRRSv uses complex strategies to evade both the innate and adaptive immune responses [6]. Because these immune evasion mechanisms are not fully understood, a sustainable treatment is difficult to find. The ease with which PRRSv moves from farm to farm further complicates control strategies [7]. In addition, the virus is genetically highly heterologous and vaccination based on a single PRRSv strain is not necessarily sufficient to protect against other strains [6].

One way to minimize the economic loss caused by PRRS is to improve disease resistance of the host. The PRRS Host Genetics Consortium (PHGC) was founded to examine the genetic basis of host responses to PRRS and understand its overall impact on pig health and growth [8]. As part of the PHGC, infection trials are conducted on approximately 200 weanling pigs each. All pigs in these studies are infected with PRRSv, and weight gain and viremia levels are measured on 0, 4, 7, 11, 14, 19/21, 28, 35 days post infection (dpi) to day 40/42, when the trial is terminated. In 2012, using a genome-wide association study on the first three infection trials, Boddicker et al. [9] reported a quantitative trait locus (QTL) on chromosome 4 (SSC4) that explained a large proportion of the genetic variance for viral load and, to a lesser extent, weight gain. In that region on SSC4, the single nucleotide polymorphism (SNP) marker WUR10000125 (WUR) on the porcine 60 K SNP chip was shown to capture most of the effect in this region. The effect of the SSC4 region, and of WUR in particular, was successfully validated in additional trials on animals with a different genetic background [10, 11]. For WUR, the B allele is the favorable allele when compared to the A allele, but the B allele has a low frequency in these challenge populations. Fortunately, the SNP marker works in a dominant manner, giving similar protective phenotypes for AB and BB animals [9].

In the last couple of years, several porcine gene expression studies, primarily at the cell culture infection level, have been executed in an attempt to unravel the porcine immune responses evoked by the PRRS virus. Of these whole genome PRRS expression studies, some focused on expression differences as response to virus strains with different pathogenicities [12, 13], others calculated expression differences between infected and control (uninfected) pigs [14–16] or *in vitro* between infected and control cells [17], and two measured whole genome expression differences due to breed [18, 19]. These latter experiments compared breeds that are more resistant to PRRS with breeds that are more susceptible to it, and were focused on understanding immunological differences to explain phenotypic differences. Thus, these experiments reported on gene expression in dissected tissue, a method that would be difficult and costly to implement in a practical selection process. In the current PHGC gene expression study, all animals were infected and comparisons were made between susceptible and more resistant pigs within breed. The difference in susceptibility was indirectly measured by growth rate post-infection and viremia levels in the blood. Bates et al. [20] earlier reported a similar study using infected susceptible pigs, that showed a high PRRSv burden (high responders, H) and infected but tolerant or resistant animals with a low PRRSv burden (low responders, L). At 14 dpi, lungs and bronchial lymph nodes were collected and several genes such as CCAAT/enhancer-binding δ protein (CEBPδ) and thioredoxin-interacting protein (TXNIP), with a differential expression (DE) level between H and L in one or both tissues, were found using the 13 K Qiagen-NRSP8 porcine oligo array [20]. A follow-up study using the 20 K Pigoligoarray on the same samples confirmed the DE of several of these candidate genes, as well as additional immune response candidate genes [21].

In gene expression studies conducted on pigs within the PHGC trials, the blood transcriptome is examined because of its collection ease, the large number of animals that can be sampled, the possibility of repeated sampling of the same individual, and, ultimately, the chance to develop biomarkers for selection purposes. In 2013, an initial gene expression study performed on the first PHGC trial was published [22]. In this study, twelve animals that represented all four combinations of two extreme phenotypes regarding weight gain (high growth rate versus low growth rate) and viral load (high viral load versus low viral load) were selected for study. The blood transcriptome of these twelve pigs on several dpi was compared using the Pigoligoarray [23], the annotation used was the current NCBI RefSeq annotation. Array probes and their annotation can be downloaded from www.animalgenome.org/pig/projects/oligoAnnot/2014/ (see GPL7435 array). One main goal of the Arceo et al. [22] study was to determine an adequate sample size and to decide which dpi were the most informative with regard to future PRRS response expression studies in the blood. The study described herein is an expansion to that study, investigating the expression profile of sufficient numbers of blood samples as proposed by Arceo et al. [22]. Samples were taken at three early time points (0, 4 and 7 dpi) for at least 20 infected animals per phenotypic group. A linear modeling approach was used to find DE genes between phenotypic extremes or different WUR genotypes. Besides annotation analyses of DE gene lists, weighted gene co-expression network analyses (WGCNA) [24] and partial correlation and information theory (PCIT) [25] were used to explore these expression datasets. Co-expression changes that look at how clustering or correlations change in response to treatment are more sensitive to detect pathway signaling differences between such treatments. In this way, co-expression analyses may be more sensitive at detecting biologically interesting effects than differential expression analyses. The WGCNA and PCIT approaches allow us to go beyond lists of individual DE genes and identify gene expression networks correlated with relevant phenotypes or WUR genotype.

## Methods

### Study design and phenotypic groups

**Fig. 1:**
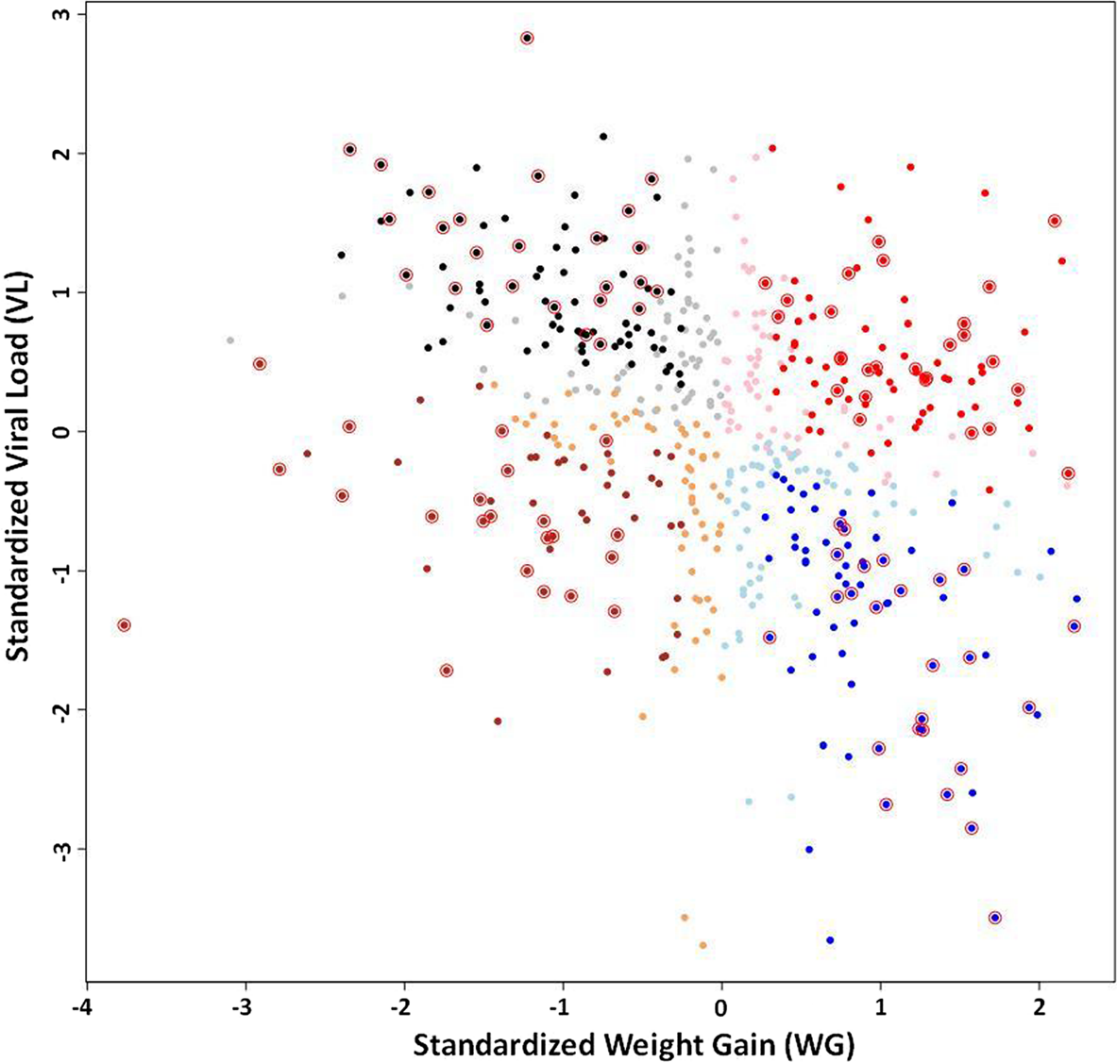
Scatterplot of PHGC1, 3, 4 animal phenotypes as shown as a function of WG and VL. Each symbol represents a pig. There are 598 pigs in total. The four phenotypic groups (LvHg, HvHg, LvLg and HvLg) are represented by a different color. The values for WG and VL are the residuals after correction for trial. The extreme animals for each group are marked with a dark color. The 100 animals selected for the microarray study are marked by circles

This study was conducted as part of the PHGC project. Experimental design, details of the infection and tissue collection procedures are described in Lunney et al. [8] and Rowland et al. [26]. Briefly, in each PHGC trial, approximately 200 pigs were transported at weaning age to the biosecure testing facility at Kansas State University and allocated in pens of 10 to 15 pigs per pen. All animals came from PRRSv, *Mycoplasma hyopneumonia*e and swine influenza virus free high health farms. After a one-week acclimation, pigs were intramuscularly and intranasally infected with a known isolate of PRRSv (10^5^ TCID_50_ of NVSL 97–7985). Blood samples were taken at 0, 4, 7, 11, 14, 19/21, 28, 35 and 40/42 dpi. Viremia levels on these dpi were measured in the serum using qRT-PCR, as described by Boddicker et al. [9]. Weight was measured at day 0 and weekly thereafter. Pigs were euthanized at 42 dpi. Viral load (VL) was measured as area under the log curve of these viremia levels from 0 to 21 dpi. Weight gain (WG) was measured from 0 to 42 dpi. Animals used in this study were part of trials 1, 3 and 4. Pigs used in PHGC trial 1 (PHGC1) and PHGC3 were the offspring of Landrace boars and Large White sows, and the average viremia levels on 4, 7, 11, 14 and 21dpi, VL over 21dpi and WG over 21 and 42dpi of these animals can be found in Boddicker et al. [9]. Pigs in PHGC4 came from Duroc sires crossed with Large White/Landrace/Yorkshire sows. Additional details on VL and WG for these animals is reported in Boddicker et al. [10]. Animals were assigned to four phenotypic groups according to VL and WG, as described by Arceo et al. [22], with minor variation: the criteria for selection was that the normalized weight and viral load were larger than 0.25 standard deviations (SD) of the mean instead of 0.5 SD of the mean. The groups were defined as follows: high VL with maximal WG, referred to as HvHg; high VL with reduced WG or HvLg; low VL with maximal WG, or LvHg; and low VL with reduced WG, or LvLg (Fig. 1). An overview of the animals used is given in Table 1. Besides collecting blood for viremia measurements, 3 mL of blood samples were also collected into Tempus™ Blood RNA tubes (Life Technologies, Carlsbad, CA, USA) at 0, 4, and 7dpi. Total RNA was extracted using Tempus™ Spin RNA Isolation Kit (Life Technologies, Carlsbad, CA, USA) according to the manufacturer’s protocol. RNA concentration was quantified using a NanoDrop ND-1000 spectrophotometer (Nano-Drop Technologies, Wilmington, DE, USA) and RNA quality was assessed using an Agilent Bioanalyzer 2100 (Agilent Technologies, Inc., Santa Clara, CA, USA).

**Table 1:** Number of animals used in this study and their phenotypes

Trial	# of animals per group				# of animals per WUR genotype		
	HvHg	HvLg	LvHg	LvLg	AA	AB	BB
PHGC 1	10	10	9	8	26	11	0
PHGC 3	9	9	10	8	21	14	1
PHGC 4	8	7	6	6	24	3	0
Total	27	26	25	22	71	28	1

Numbers are given for each PHGC trial separately. Groups are formed according to viral load and weight gain (HvHg, HvLg, LvHg and LvLg); genotypes are AA, AB and BB for the WUR10000125 SNP marker

### Ethical statement

The study was approved by the Kansas State University Institutional Animal Care and Use Committee (IACUC).

### Microarray design and analysis

RNA samples (0, 4 and 7dpi) were reverse transcribed using the Amino Allyl MessageAmp II aRNA Amplification Kit (Ambion/Life Technologies), labeled with N-hydroxysuccinate (NHS) ester Cy3 or Cy5 dyes (GE Healthcare, CA, USA), and hybridized to a previously described 20 K 70-mer oligonucleotide microarray, named the Pigoligoarray [23]. A block reference design [27] was followed to allocate samples to slides with each individual pig’s 0dpi-sample serving as reference for the other two samples from the same animal. Reference sample dye flipping was performed across replicates within phenotypic groups to allow separation of dye and 0dpi effects [27]. Fluorescent images and fluorescence intensity data were collected as previously described [22, 23]. Median intensities were background corrected with Normexp method fixing the offset parameter κ = 50 [28]. Background corrected data was normalized using a within print-tip loess-location normalization [29]. All computations were implemented in R [30] through LIMMA [31]. Normalized log-ratios of 4dpi-0dpi and 7dpi-0dpi were analyzed separately. A linear model, accounting for dye, array, trial, group and WUR SNP genotype was fit on a spot oligonucleotide basis [32] using the LIMMA program [31]. Several contrasts were computed: 1) WUR genotypes, 2) interaction between growth and viral load groups, 3) effect of viral load within growth group, 4) effect of growth within viral load group and 5) 4dpi or 7dpi versus 0 dpi. To account for multiple testing, the false discovery rate (FDR: q-value) procedure [33] was used to adjust p-values obtained for each contrast.

### Validation of the microarray results using RNAseq

Validation of DE gene lists of genes with a *q*-value ≤ 0.05 was done using log normalized and model adjusted expression values obtained from an RNAseq experiment [34, 35]. In short, the RNAseq experiment was performed on 16 animals of PHGC3, 7 of which were among the 100 animals used in the microarray experiment, the expression profiles of the remaining 9 animals are independent. To normalize the RNAseq data, the Trimmed Mean of M-values (TMM) method was used (edgeR package version 3.4.2). Data for lowly expressed genes, whose maximum TMM expression value across samples was less than 10, were removed, and model adjustments were made for pre- and post-globin reduction RNA Integrity Number, and 5′-3′ read skewness. In all, 8,997 annotated genes were retained. For the 4dpi-0dpi microarray dataset, 31 of the 67 genes from the DE list at a q-value of ≤ 0.05 were among those 8,997 genes. For the 7dpi-0dpi, 15 of 34 were annotated in both the microarray and RNAseq experiment. Those common 31 and 15 genes were used to test validation of the 4dpi-0dpi and 7dpi-0dpi microarray experiment, respectively.

### WGCNA analysis and module stability

The WGCNA R package was used to cluster highly correlated genes and find clusters whose expression was correlated with the traits examined [24]. WGCNA was carried out on data from all 19,981 oligonucleotides of the 4dpi-0dpi dataset for all 100 animals. An adjacency matrix based on expression correlation was created using a soft threshold procedure to allow a scale free topology. The clusters created by WGCNA are called modules, and the minimum number of genes in a module was set to 30. Genes not classified in a correlated module were grouped in a grey ‘rest of data’ module. To see whether modules were stable for each dataset, the module stability was examined using the module stability analysis embedded in the WGCNA package [36].

Once the modules were created, the animals’ phenotypic information was correlated with the module eigengene (ME). The eigengene of a module is defined as the eigenvector associated with the first principal component of the expression matrix and is used as a ‘supergene’ or a linear combination of expression from all genes in the module [37]. Phenotypes to analyze in our experiments were WG, VL and WUR genotype. For WG and VL, the raw values were adjusted for trial mean effects by computing the residuals of a linear model that included the categorical effect of trial. These adjusted values will be referred to as WG residual and VL residual. Because the desired values of WG (higher) and VL (lower) are opposite in direction, a Desirability coefficient (Des coef) was calculated to combine the WG and VL variables as follows: $\frac{WG-\overline{WG}}{stdev(WG)}-\frac{VL-\overline{VL}}{stdev(VL)}$. The WUR genotype was coded so that BB was −1, AB was 0 and AA was +1. Additionally, correlations were calculated between the MEs and weight and viremia in the serum on specific days. For weight, this was done at 0, 7, 14, 19/21, 28, 35 and 40/42 dpi, for viremia days examined were 4, 7, 11, 14 and 19/21 dpi.

### CTEN analysis

Cell type enrichment (CTEN) was used to see if WGCNA modules that were significantly correlated with a trait of interest, pointed to enrichment of one or more specific cell types to explain expression patterns of specific modules [38]. For this analysis, all available gene symbols in a module were uploaded and the list was compared to the existing CTEN database [38], which consists of highly expressed cell specific genes known for human and mouse. As output, the program uses Benjamini-Hochberg adjusted p-values to determine the significance of enriched cell types or tissues and creates color-coded figures indicating this enrichment.

### PCIT analysis

To identify potential different regulators in phenotypically divergent animals, the PCIT algorithm was run. Full details of this algorithm are described in Reverter and Chen [25] and in Koesterke et al. [39, 40]. In this study, pairwise contrasts were made between Hg and Lg animals, between Hv and Lv animals, between High Des coef (higher WG and lower VL) and Low Des coef animals, and between animals with a AA versus AB WUR genotype. Since there was only one BB animal, it was omitted in this analysis. The significance of a partial correlation between a target and hub gene was determined using an information theory approach that sets the significance threshold based on the direct and partial correlation for all tests performed in the data [25]. In this way, the significance of an edge in the network is determined by the information in a specific dataset. Only significant partial correlations were used in the differentially hubbed (DH) gene analysis. A script was written to determine the DH results that identified the hub genes [40].

### GO Term Enrichment

Throughout the experiment, the functional annotation tool DAVID Bioinformatic Resources v6.7 [41] was used to define gene ontology terms enriched by a set of genes. First, DAVID analyses were performed on DE lists created using a maximum FDR adjusted *p*-value of 0.10 as criterion [33]. Second, DAVID analyses were performed on lists of genes corresponding to significant WGCNA modules. WGCNA modules were considered significant for a certain trait when the nominal *p*-value of the correlation between the ME and the trait of interest was less than 0.10. Third, DAVID analyses were performed on the entire list of differentially wired correlates from the top 10 hub genes resulting from the PCIT analysis. Annotations were performed using the human Ensembl gene ID numbers, to maximize recognition by the DAVID tool. As a background gene dataset for these DAVID analyses, all annotated genes of the microarray were used. Enrichment scores higher than 1.3 were regarded as significant.

## Results

### The use of microarrays to analyze expression differences due to infection, between phenotypic groups and between animals having a different WUR genotype

To determine the gene expression differences among the four extreme growth and viremia phenotypes in response to PRRSv infection, we selected ~25 pigs that belonged to each of the HvHg, HvLg, LvHg or HvLg groups (Table 1, Fig. 1). This population also segregated the SSC4 QTL region that is marked by the WUR10000125 SNP [9], allowing a test of genotype effect in the same population. Animals with specific genotypes at this locus have been shown to have different infection response phenotypes in other PHGC studies [9]. For example, in the current dataset, we found that viremia levels were already significantly different between AA (1461.9 ± 175.6) and AB animals (561.1 ± 82.3) at 4dpi (*p*-value = 0.005). A microarray analysis was then performed on RNA prepared from whole blood collected at 0, 4 or 7 dpi [22].

**Fig. 2:**
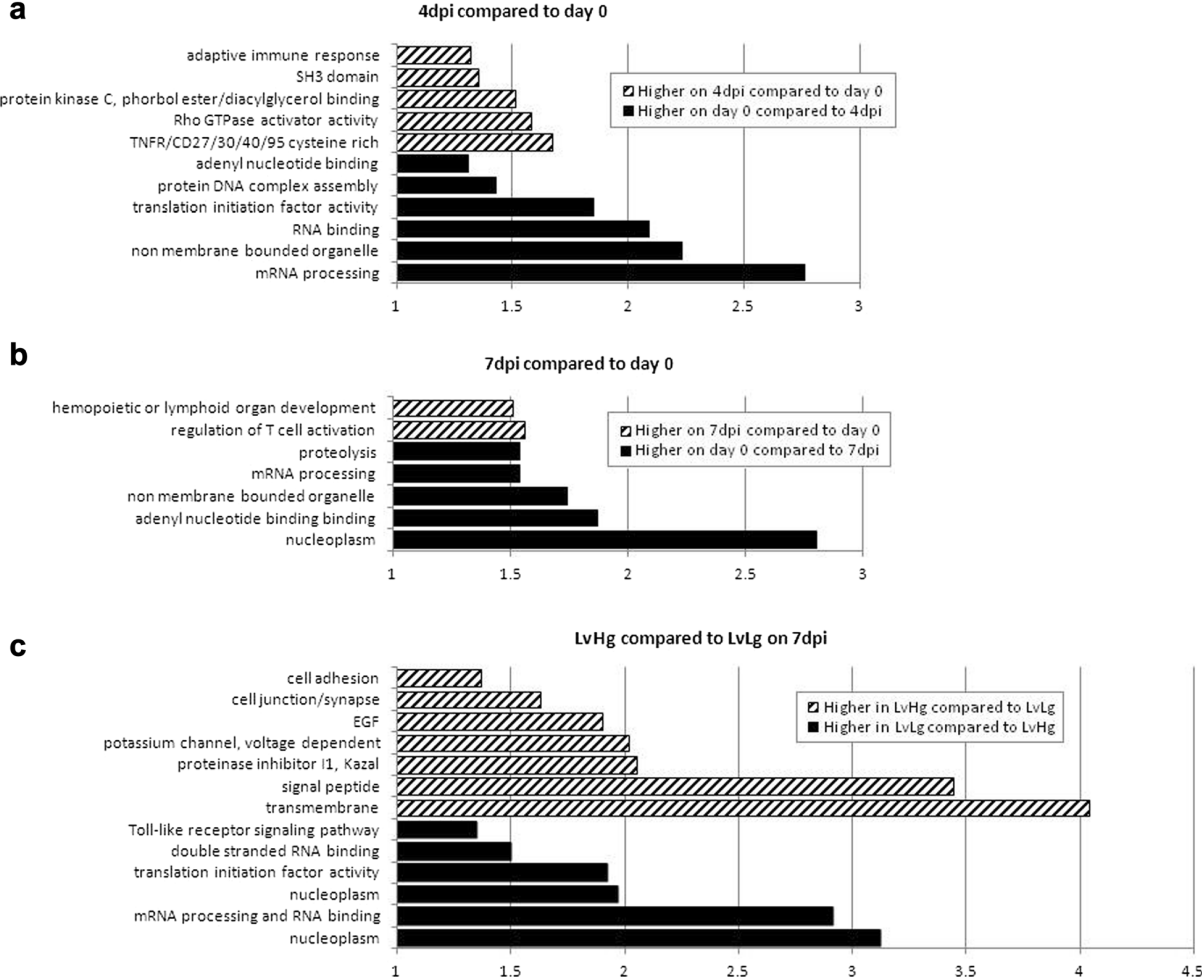
GO term annotation of DE gene lists compared between dpi. Significant clusters (Enrichment score *p*-value ≥ 1.30) are described by a given explanatory name. **a** GO terms that describe differentially enriched clusters between 4dpi and day 0 (striped bars: cluster more expressed in 4dpi data compared to day 0 data, black bars: cluster more expressed in day 0 data compared to 4dpi data). **b** GO terms that describe differentially enriched clusters between 7dpi and day 0 (striped bars: cluster more expressed in 7dpi data compared to day 0 data, black bars: cluster more expressed in day 0 data compared to 7dpi data). **c** GO terms that describe differentially enriched clusters between LvHg and LvLg animals in the 7dpi dataset (striped bars: cluster more expressed in LvHg animals compared to LvLg animals, black bars: cluster more expressed in LvLg animals compared to LvHg animals)

First, a linear model analysis was used to identify significant gene expression differences due to a PRRSv infection over time. Blood RNA levels at 4dpi versus 0dpi, or 7dpi versus 0dpi were compared. Significant DE was found in 67 and 239 genes between 4dpi and 0dpi at an FDR of 0.05 and 0.10, respectively (Table 2). The full list of DE genes is shown in Additional file 1: Table S1. For the 7dpi-0dpi dataset, 34 and 165 genes were found at an FDR of 0.05 and 0.10, respectively (Table 2, Additional file 2: Table S2). GO term analyses were performed at an FDR of 0.10. They revealed that several immune response pathways were upregulated after infection (adaptive immune response, SH3 domain, regulation of T cell activation). However, enrichment scores for these GO terms were only marginally significant and therefore did not indicate a strongly upregulated pathway (Fig. 2). mRNA binding, translation initiation factor activity and mRNA processing activities were downregulated after infection.

**Table 2:** Numbers of DE genes for the different comparisons

	Comparison	# of DE genes (FDR = 0.10)
WG effect	HvHg-HvLg contrast on 4dpi	0
	HvHg-HvLg contrast on 7dpi	0
	LvHg-LvLg contrast on 4dpi	2
	LvHg-LvLg contrast on 7dpi	709
VL effect	HvHg-LvHg contrast on 4dpi	0
	HvHg-LvHg contrast on 7dpi	0
	HvLg-LvLg contrast on 4dpi	2
	HvLg-LvLg contrast on 7dpi	0
WG and VL interaction effect	(HvHg-HvLg)-(LvHg-LvLg) contrast on 4dpi	1
	(HvHg-HvLg)-(LvHg-LvLg) contrast on 7dpi	0
day effect	4dpi-0dpi contrast	239
	7dpi-0dpi contrast	165
genotype effect	genotype (AB vs AA) contrast on 4dpi	0
	genotype (AB vs AA) contrast on 7dpi	0

Comparisons were made for a WG, VL, day and genotype effect. The FDR was set at 0.10

Second, a linear model that contrasted different phenotypic extremes or WUR genotypes was performed to find differentially expressed genes. Expression differences were investigated between Hg and Lg animals within Hv or Lv groups on both 4dpi and 7dpi. A similar analysis was done to compare Hv and Lv animals within the Hg or Lg groups. Only the comparison between LvHg and LvLg animals on 7dpi showed sufficient DE genes (709) at an FDR of 0.10 to permit annotation enrichment analysis (Table 2, Fig. 2c). GO terms showed enrichment of the Toll-like signaling pathway in this contrast but, as above, this enrichment was only marginally significant. Interaction effects between the four quadrants did not identify many differentially expressed genes on either of the days, and a similar result was seen for the WUR genotype contrast (Table 2).

### Validation of the microarray results using RNAseq data

To validate these microarray results, DE gene lists were compared with RNAseq data collected from blood samples of similarly infected animals. For the 4dpi-0dpi dataset, comparing the DE gene lists of genes with a q-value ≤ 0.05 revealed that 11 of 31 genes present in both the microarray and the RNAseq experiment were significantly up- or downregulated (q-value ≤ 0.05 for the microarray; q-value ≤ 0.10 for RNAseq) (Additional file 3: Table S3). The fold change of these genes measured by microarray and the fold change measured by RNAseq were significantly correlated overall (Pearson’s correlation of 0.42; *p*-value = 0.019). For the 7dpi-0dpi dataset, the differential expression of only 1 of 15 genes was confirmed (Additional file 3: Table S3) and the correlation between the microarray and the RNAseq data was not significant (Pearson’s correlation of −0.30; *p*-value = 0.28). Since the microarray results could not be validated for the 7dpi-0dpi dataset, further network analyses were only performed on the 4dpi-0dpi dataset.

### WGCNA analysis on 4dpi-0dpi to identify co-expressed genes whose expression pattern was significantly correlated with the examined phenotypes/genotypes

**Fig. 3:**
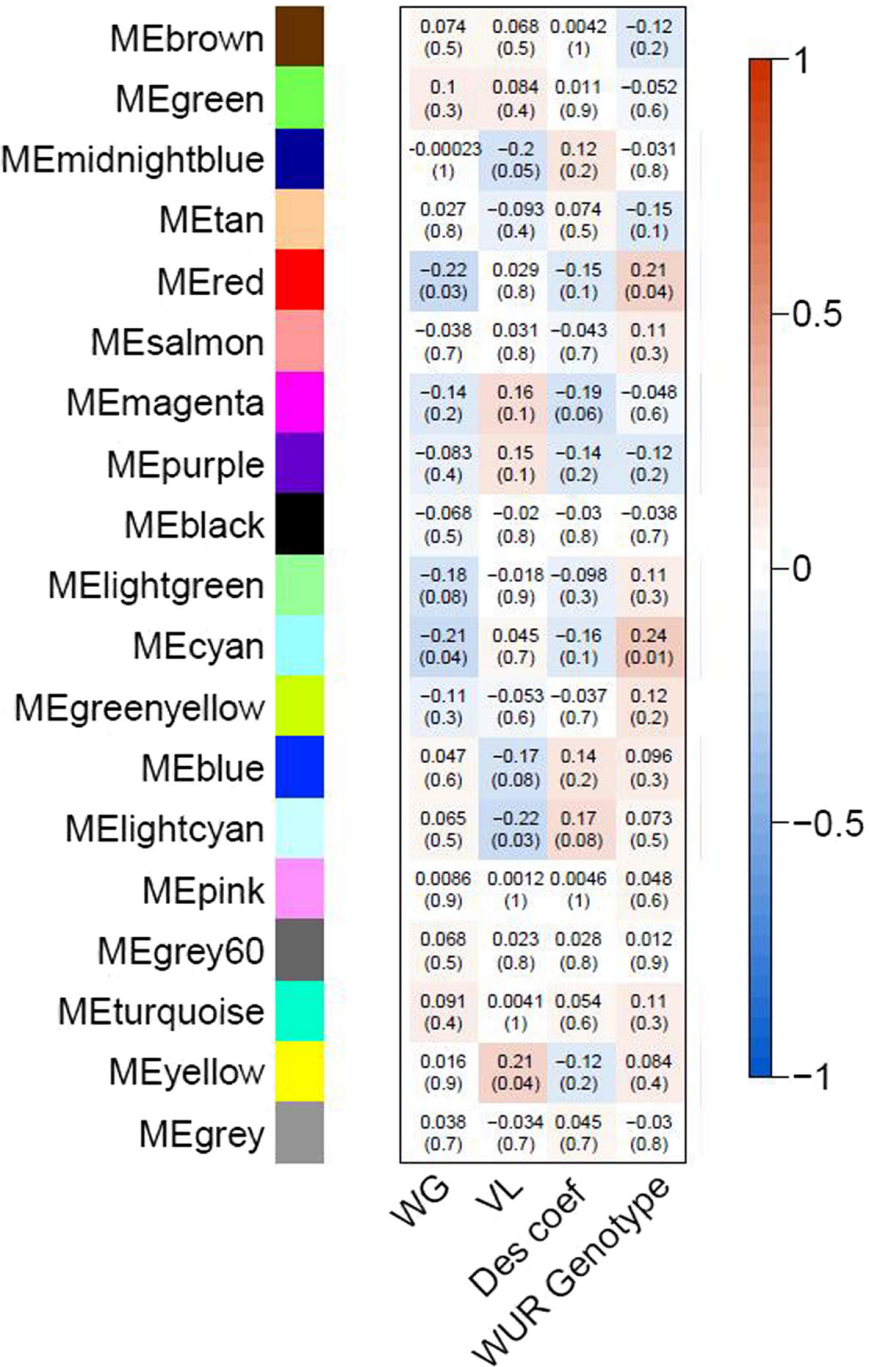
WGCNA modules and the MEs’ correlations with the traits of interest for 4dpi-0dpi comparison. Horizontally, MEs are named by the module colors, arbitrarily assigned by WGCNA. Vertically, phenotypes of interest are listed (WG, VL, Des coef and WUR genotype). Numbers given are the correlation coefficients between the respective ME and the trait of interest, with nominal *p*-value between brackets. The more intensely blue a box is colored, the more negatively its correlation is with that trait, the more intensely red, the more positive the correlation with that trait

Because the one-gene-at-a-time analysis using a linear model failed to provide biological insight into the differences between pigs with extreme phenotypes after PRRSv infection, the focus then was on clusters of genes with a similar expression pattern rather than individual gene expression values. For this approach, the clustering tool WGCNA was used to analyze the 4dpi-0dpi dataset. To create the WGCNA modules, a soft threshold power of 3 was chosen, as suggested by the software as being the best threshold to create a scale free topology, while still giving a suitable amount of node connectivity. WGCNA created 18 modules based on the correlation of 4dpi-0dpi expression ratios across the 100 samples and they were assigned a color label. All uncorrelated genes were assigned to a grey module (Fig. 3). The correlations of all MEs with trial were examined and shown to be non-significant, as expected in trial-corrected data (data not shown). The correlations of MEs with WG and WG residuals or with VL and VL residuals were similar, so only correlations with WG and VL are further discussed. Four modules had MEs that were significantly correlated (*p*-value < 0.1) with VL (midnightblue, blue, lightcyan and yellow), while three MEs were significantly correlated with WG (red, lightgreen and cyan). Two of those three (red and cyan) were significantly correlated with WUR genotype as well. Furthermore, Additional file 4: Figure S1 shows the correlations between these modules and weight or serum viremia on specific days. Most of the modules that were significantly correlated with an overall WG or VL show particular time points that indicate a strong association with the gene expression patterns.

To evaluate the stability of these modules, networks were created with random subsets of the original samples (Additional file 5: Figure S2). Overall, the modules were consistent across all datasets created. BioLayout Express 3D (BE3D) was used to visualize the spatial relationship between the modules and their genes (Additional file 6: Figure S3) [42]. Genes whose individual expression profiles are correlated with at least one other gene at a Pearson correlation of *r* = 0.70 are shown. The gene nodes were colored according to the module color assigned by WGCNA (Fig. 3).

### Annotation of the modules created by WGCNA

To determine the biological relevance of these correlations, GO enrichment was examined in gene lists of modules whose ME was significantly correlated with traits of interest. Additional file 7: Table S4 gives an overview of the enriched GO terms of these module gene lists, the number of genes in each, the number of available human Ensembl gene IDs for those genes and how many of them were recognized by DAVID. An explanatory name was chosen to describe the cluster based on the GO terms enriched.

When performing a WGCNA analysis on the 4dpi-0dpi dataset, a total of 7 modules were significantly correlated with VL or WG (*p*-value < 0.1). GO enrichment analysis for modules significantly correlated with VL was not very informative. The blue module (*p*-value = 0.08), which contained over 2300 genes, was enriched in annotation for very broad terms such as transmembrane, amino acid transmembrane transporter and ion transport. Analysis of other significant modules for VL (midnightblue: *p*-value = 0.05, lightcyan: *p*-value = 0.03 and yellow: *p*-value = 0.04) also did not reveal explicit pathways. For WG, the modules that were significantly correlated with the trait were all negatively correlated (lightgreen: *p*-value = 0.08, cyan: *p*-value = 0.0 and red: *p*-value = 0.03), indicating that the overall expression of these genes was higher in the animals having a lower WG. Two modules whose ME was significantly correlated with WUR genotype (cyan: *p*-value = 0.01; red: *p*-value = 0.04), had a ME that was also correlated with WG, in a direction that indicates that AB animals have a desirable PRRS response phenotype (higher weight gain post-infection) when compared to AA animals, as was discovered by Boddicker et al. [9]. The light green module, which contained only 49 genes, did not show any significant enrichment. The cyan module contained 127 genes and was annotated as a module important for RNA processing, chromosomal organization and DNA replication. The red module, containing 506 genes, was most immunologically relevant, with GO terms such as innate immune response, Toll-like receptor signaling pathway and complement activation.

**Fig. 4:**
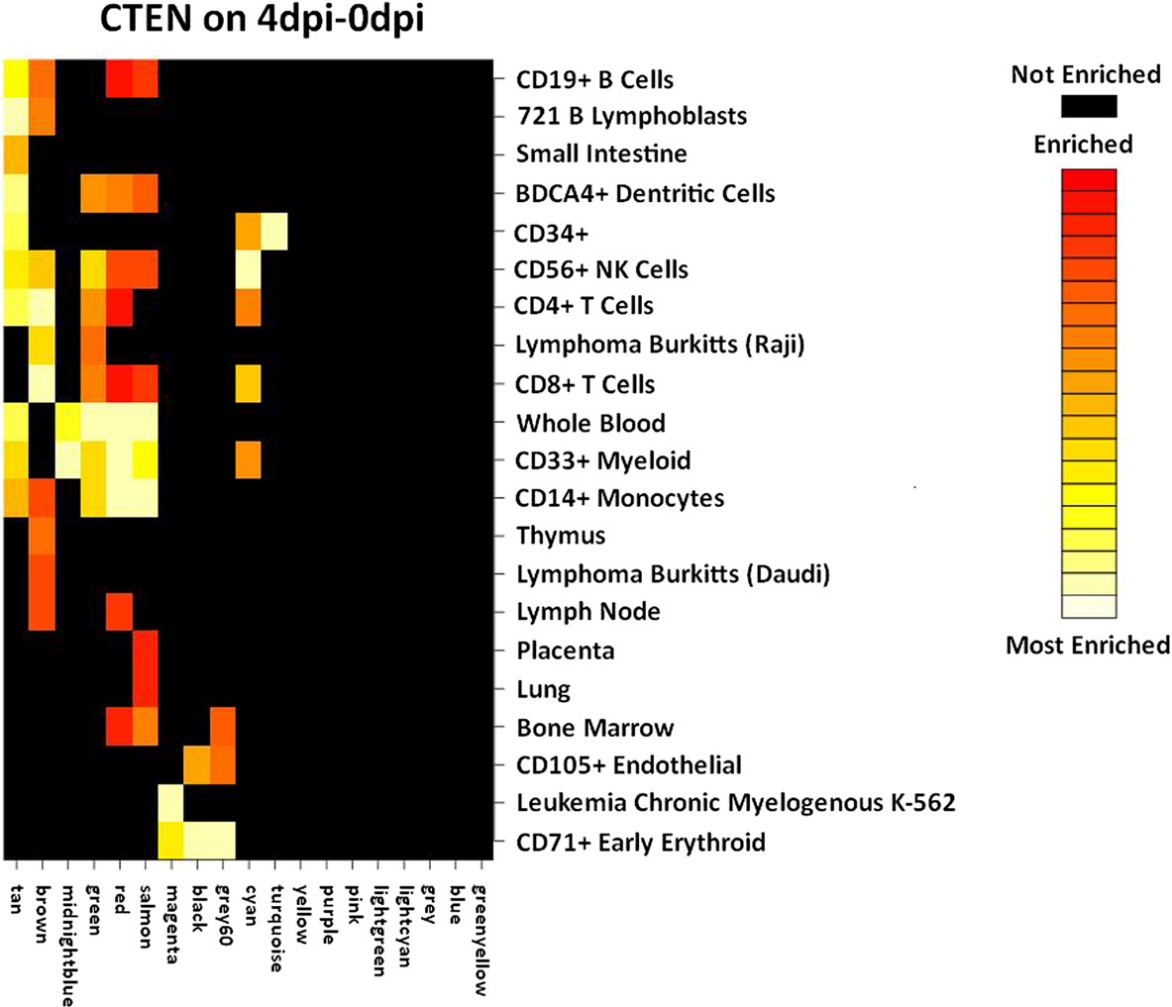
Cell Type Enrichment (CTEN) analyses on the 4dpi-0dpi dataset. The WGCNA clusters are noted along the abscissa. Black squares represent no enrichment in cell type. From red to yellow to white are all significant enriched cell-type specific genes within each module gene list (enrichment score ≥ 2.0), going from enriched to most enriched

To see if a significant correlation of the modules was primarily due to an up- or downregulation of expression of genes in that cluster, or whether it could be partly explained by specific cell types, a cell type enrichment analysis was performed on both datasets using the web-based tool CTEN (Fig. 4). Eleven modules showed enrichment for gene expression patterns observed for specific cell types (*p* ≤ 0.01). Within these, the midnightblue, red and cyan modules were significant for a trait of interest, as mentioned earlier.

### PCIT analysis on 4dpi-0dpi to find regulatory differences between phenotypic groups and between animals having a different WUR genotype

**Fig. 5:**
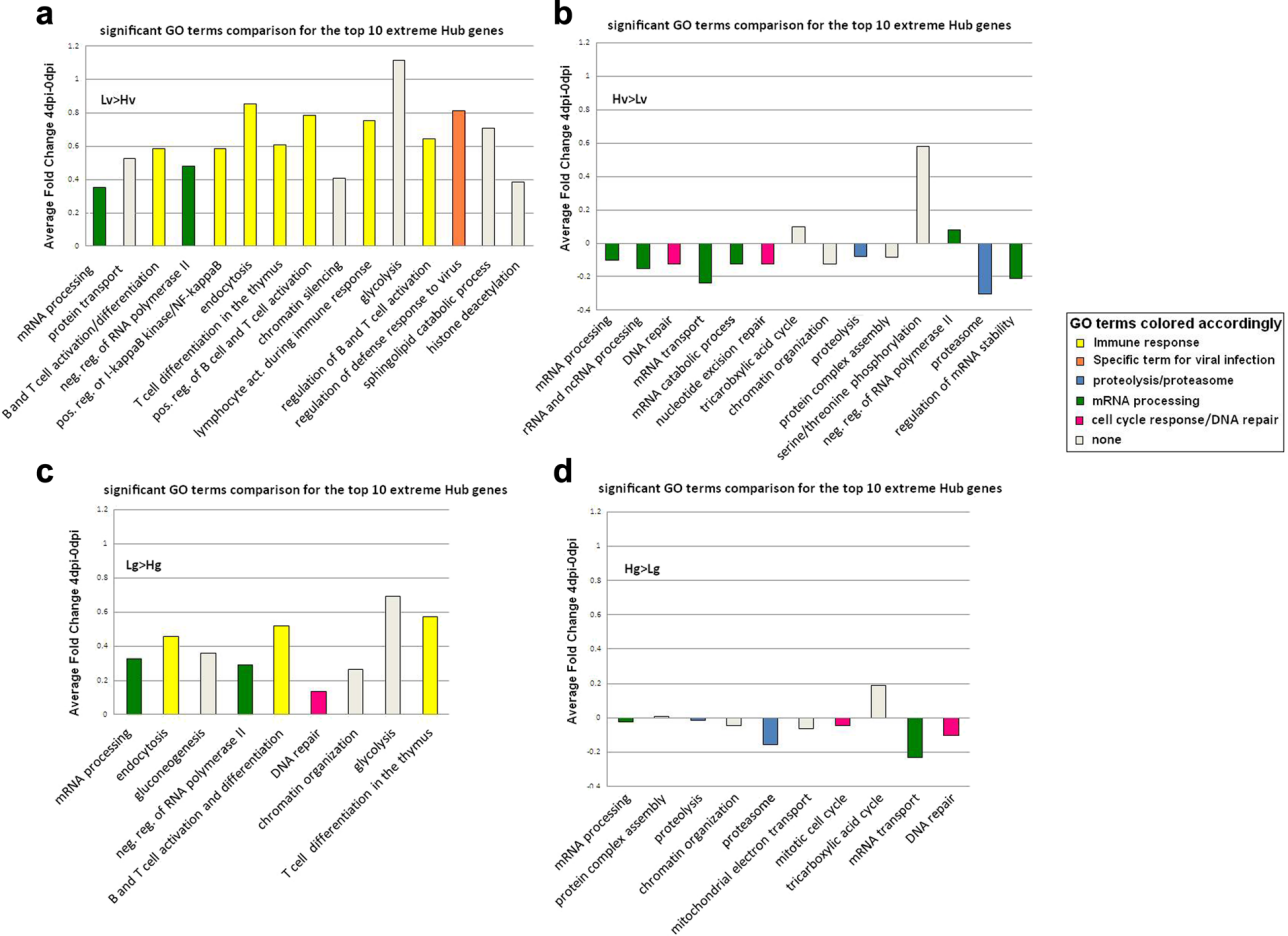
GO term enrichment for correlates of hub genes that contrast VL and WG. These hub genes are the top 10 genes with the most extreme wiring between VL (**a** and **b**) and WG (**c** and **d**) groups according to the PCIT analyses. On the Y axis, the average log_2_ fold change of the correlates that belong to a GO term is shown. Colors represent specific GO term groups as shown in the legend

**Fig. 6:**
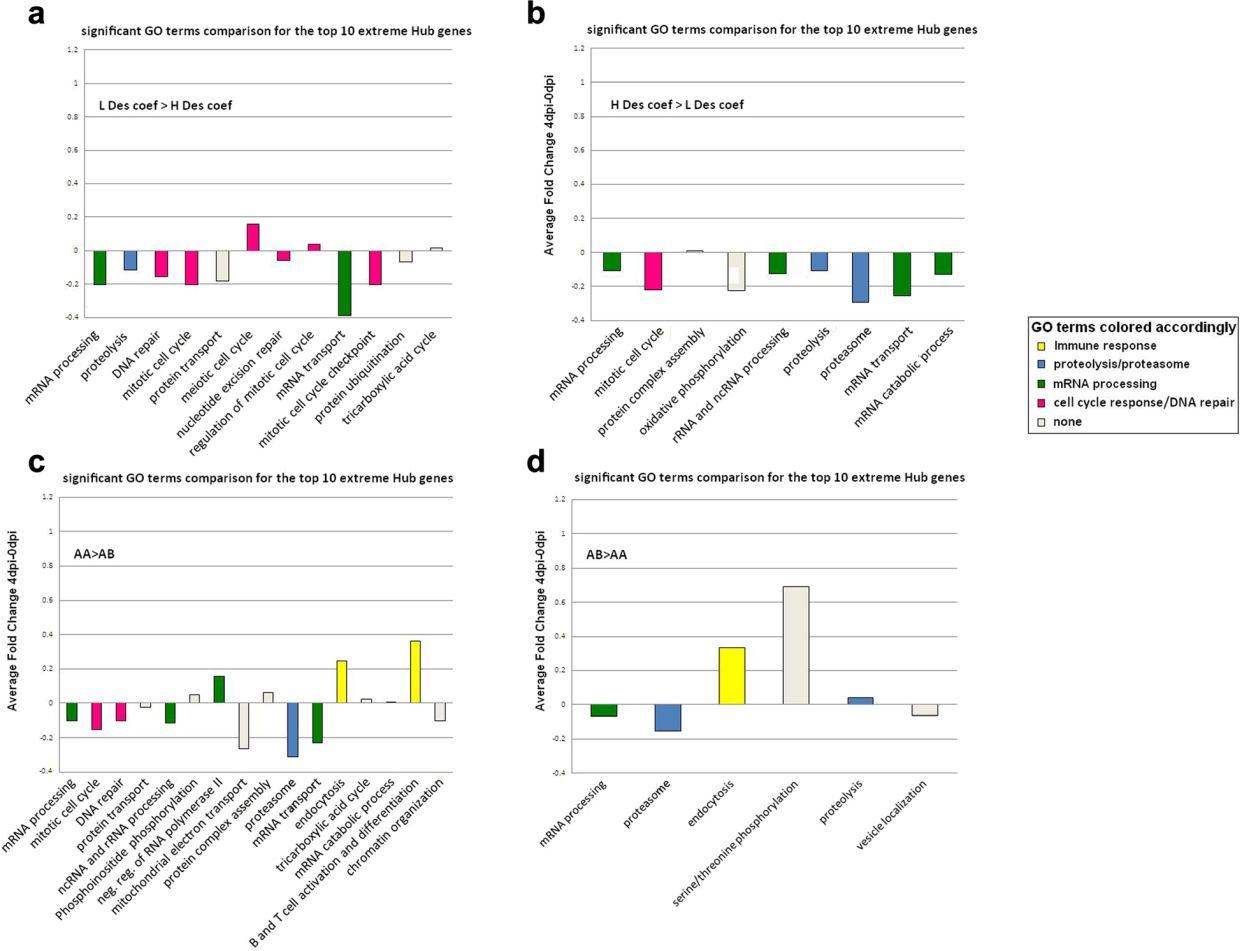
GO term enrichment for correlates of hub genes that contrast Des coef and WUR genotype. These hub genes are the top 10 genes with the most extreme wiring between Des coef (**a** and **b**) and WUR genotype (**c** and **d**) groups according to the PCIT analyses. On the Y axis, the average fold change of the correlates belonging to a GO term is shown. Colors represent specific GO term groups as shown in the legend

A PCIT analysis explores differences in connectivity strength between gene expression patterns in two contrasting groups of animals, as measured by connections drawn between genes in a correlation network. Genes shown to have significant differences in connections between groups with a distinct set of genes are called hub genes and could identify a difference in gene regulation between these groups. In this study, the phenotypic contrasts provided to PCIT were Hg versus Lg, Hv versus Lv, High Des coef (>0.5) versus Low Des coef (<0.5), and AA versus AB WUR genotype. Additional file 8: Table S5 shows for each contrast the top 10 differentially connected hub genes, their annotation and the difference in number of correlates between the two contrasts. For these PCIT analyses, Information Theory was used to determine the significance of correlation in these PCIT analyses. This approach considers the total number of correlations calculated for the entire dataset and only the partial correlation values that achieve the significance threshold for the entire dataset are retained to define the connectivity between a target and hub gene. To explore the effect of these differences, all correlates of the 10 extreme DH genes were combined and the resulting gene list examined for GO term enrichment. In Fig. 5a, significant GO annotation terms are shown for the correlates that were only present in the Lv group or more strongly connected to at least one of the top 10 extreme hub genes in the Lv group when compared to the Hv group. The height of the bars is the average log_2_ fold change between 4dpi and 0dpi of all correlates annotated with the respective GO term. Figure 5b shows the enriched annotation of correlates of the hub genes in the Hv group. Several immune-related pathways were significantly over-represented in the Lv group compared to the Hv group. Similar results for GO terms of correlates tightly connected to the Lg and Hg group are shown in Fig. 5c and d, with more enrichment of immune-related pathways in the Lg group compared to the Hg group. Animals with a low Des coef (*n* = 34) were contrasted with the animals with a high Des coef (*n* = 38) in Fig. 6a and b. The GO terms enriched for the correlates of the top 10 hub genes in AA and AB animals is shown in Fig. 6c and d. For these last two contrasts, GO terms did not reveal major correlation network differences between more and less favorable animals.

## Discussion

In this study, systemic differences among 100 animals that exhibited diverse responses to PRRSv infection were explored using microarray analysis of whole genome blood expression levels. Although a substantial number of animals that represented each of the four extreme phenotypic groups were evaluated in a linear model based approach, few differentially expressed genes were found. The linear model declared a relatively small number of genes upregulated at 4dpi and 7dpi when compared to 0dpi, while DE genes were found in a single phenotypic group comparison, and this only when looking at the 7dpi expression profiles. GO annotation enrichment analyses of these lists did illustrate some upregulated immune responses after infection for both 4dpi and 7dpi, and in the Lg group compared to the Hg group at 7dpi. Nevertheless, this analysis found few terms were enriched within these gene lists, and therefore a limited amount of biological insight could be gleaned from these analyses.

In addition, after trying to validate the gene expression pattern differences measured by the microarray between 4dpi or 7dpi and 0dpi, it became clear that the 7dpi-0dpi dataset was difficult to validate, as was also seen earlier by Arceo et al. [22]. To validate these data, RNAseq data from blood taken from an independent set of animals was used. Although both RNAseq and microarray analyses measure the transcriptome, studies report different genes as DE using one method or the other, even when using the exact same samples [43–45]. In addition, the difficult validation of the 7dpi dataset may also be due to the fact that different groups of animals were analyzed by the two techniques, and at 7dpi, the virus is still replicating in some animals while in others the adaptive immune response might already be ramping up. Thus, significant variation is likely present in the response to PRRSv observed at 7dpi across experimental groups. For these reasons, we decided to only examine the 4dpi-0dpi dataset in further analyses.

The main goal of this study was not to investigate differences due to infection, but to explore whether there were differences between the phenotypic groups that diverged after infection. Therefore, other more sensitive analyses were performed. These more sensitive co-expression analyses emphasize differences in gene network up- or downregulation rather than individual gene expression differences [46].

### The red module is related to inflammation and its average expression is negatively correlated with weight gain after a PRRSv infection

The red module, with 506 genes, was enriched for several immune response terms and could therefore contain genes of great importance in a PRRSv infection. An overview of the genes in the red module at 4dpi-0dpi is shown in Additional file 9: Table S6. The CTEN analysis revealed that the red module genes were enriched in genes specifically expressed in CD33+ myeloid cells and CD14+ monocytes. This could indicate that the expression patterns of the genes in this module were not solely due to transcriptional changes but possibly also due to a difference in monocyte recruitment into the blood. It has been described that PRRSv infection causes an increase in CD14+ expression throughout the early stage of infection, due to a rise in CD14+ monocytes that differentiate to macrophages and migrate to bronchoalveolar spaces [47]. The red module contained several anti-apoptosis members of the BCL2 family (*BCL2A1*, *BCL2L14*, *BCL2L15*, *MCL1*) earlier reported to be upregulated after PRRSv infection [14, 15]. Anti-apoptosis is often seen in the early stage of a PRRSv infection: this is beneficial for the virus since anti-apoptosis expands time for viral replication in macrophages [48, 49]. Only later in the infection do PRRSv infected macrophages die by apoptosis [48]. A large portion of genes present in the red module encodes pathogen-recognition receptors (PRRs) such as Toll-like receptors (*TLR2*, *TLR4*) and the CD14 co-receptor for TLR4 (*CD14*), C-type lectin receptors (*CLEC2B*, *CLEC2D*, *CLEC4E*, *CLEC7A*) and RIG-1-like receptors (*DDX58* or *RIG1*, *MDA5* or *IFIH1*). PRRs play a main role in triggering inflammatory responses by activating and inducing release of cytokines, chemokines and type I interferons (IFNs) [50]. Additionally, several members of this module are known as interferon-stimulated genes (*OAS1*, *RNASEL*, *MB21D1*, *BST2*, *IFIT3*, *ISG20*, *RSAD2*, *DDX60*, *USP18*) [51]. Many of these genes where also found in the type I interferon/cytokine mediated immune response cluster found by the Immune Response Annotation Group [52]. However, genes, seen to have a specific PRRS response expression pattern, elicited by macrophages or lymph nodes, were not clustered in this red module, nor in any other cluster. Type I interferons induce an innate antiviral response, and PRRSv infection has been observed to dampen or delay the type I interferon transcriptional response [19, 53, 54]. Ait-Ali et al. [19] described a difference in PRRS susceptibility between macrophages isolated from Landrace versus Piétrain pigs. The main difference was the rate with which type I interferon transcriptional changes occurred: they suggested this as the underlying reason for breed differences in PRRS susceptibility [19]. Souza et al. [55] reported that IFNα levels in serum increased rapidly after a PRRS infection. However, after 4dpi, the best animals (LvHg group) had the fastest return to basal levels; as early as 7dpi, the IFNα levels were significantly lower in this group compared to other phenotype quadrants [55]. In our study, the red module, that contained several interferon-stimulated genes, was found to be negatively correlated with WG and positively with WUR genotype, which meant that animals with a lower weight at 42dpi or animals with the unfavorable genotype for WUR expressed higher levels of these genes at 4dpi. Because a significant difference in viremia was noticeable between the two WUR genotype groups already at 4dpi in these pigs, we propose that the effect of genotype on phenotype is already present by this time. Since the red module was positively correlated with viremia at 4dpi, our hypothesis is that the resistant AB animals control virus replication through red module interferon-stimulated genes prior to 4dpi. This earlier, and possibly more effective response, is supported by IFNα data on similarly challenged PHGC cohorts, which shows that LvHg animals more quickly bring down their IFNα serum levels. This drop is significantly faster and to lower levels for these LvHg animals compared to the other groups, indicating a faster initiation of the resolution of the anti-viral response [55]. Hulst et al. [56] showed that type I interferon expression in the blood can differ between susceptible and more resistant pigs to a classical swine fever infection. In their study, no significant pathways involved in the induction of the antiviral IFN type I response were upregulated in chronically diseased pigs at 4dpi or 8dpi, while in pigs that recovered rapidly, these pathways were already significantly upregulated at 4dpi until recovery at 10-12dpi [56], which supports our theory that better adapted animals induce the interferon type I response faster. We further speculate that susceptible AA animals demand more energy by keeping their immune system active longer (higher expression in blood of red module genes at 4 dpi), which could manifest in poor growth in the long run [57].

Besides interferon-stimulated genes, other inflammatory response genes were found in the red module. Miller et al. [13] recently described the infection response in tracheobronchial lymph nodes to two PRRSv strains, a Chinese highly pathogenic rJXwn06 strain and the US VR-2332 strain. They found that the RNAs for serum amyloid A2 acute phase proteins, *S100A8*, *S100A9* and *S100A12*, were amongst the top upregulated genes in animals inoculated with these strains compared to sham inoculated pigs [13]. *S100A8* and *s100A12* are found in the red module. Another gene in this module was *TREM1*, known to trigger release of pro-inflammatory chemokines and cytokines, and found by Miller et al. [13] and Badaoui et al. [12] to be highly induced after PRRSv infection. Furthermore, *NLRP3*, *CASP1*, *IL1B* and *IL18* were also found in the red module. The inflammasome gene *NLRP3* is known to activate *CASP1*, which leads to the activation of *IL1B* and *IL18* [58]; all have been previously shown to be upregulated after PRRSv infection [59]. These inflammatory responses are of interest with regard to the different WUR genotypes, since the region near the WUR SNP in the SSC4 region contains several immune-related genes such as members of the Leucine Rich Repeat Containing protein 8 (*LRRC8*) family [60] and genes belonging to the Guanylate Binding Protein (*GBP*) family [61]. Recently, a putative causal mutation for the SSC4 PRRS host response QTL associated with WUR was identified in *GBP5* [35]. Since *GBP5* directly regulates *NLRP3* inflammasome assembly, it is interesting that the red module includes *NLRP3* and several of the inflammatory genes that it regulates. Finally, Wysocki et al. [21] have described the importance of the complement cascade during PRRSv response, and the red module contains genes encoding several complement regulatory proteins such as C4BPA, the decay-accelerating factor (DAF) CD55, the membrane cofactor of proteolysis (MCP) CD46 and the C1 inhibitor SERPING1, all inhibitors of complement activation [62]. This points to a difference in complement regulation in the slower growing animals, or those that have the unfavorable WUR genotype.

### PCIT analysis clearly indicates immune network differences between phenotypic divergent animals early after PRRSv infection

In a next step, PCIT was used to explore the regulatory networks that changed between animals with different phenotypes or genotypes at 4dpi. The largest difference in GO terms enriched in the group of correlates could be seen in those for lymphocyte activation, and this effect was strongest when the viral load phenotypes were contrasted. In other words, animals that belonged to the Lv group had top hub genes with tighter connections to genes in the immune activation pathways than did the Hv group at 4dpi. This difference was also noticeable when looking at the GO term enrichment of the correlates of the 10 extreme hub genes in the Hg and Lg groups, where animals showed tighter connections to immune genes in the Lg group. As discussed previously, this result can be explained by the allocation of energy to immune response activation rather than growth. Thus, the Lg animals may partition more energy to immune response and have less energy to put towards weight gain. The difference in connection to the immune genes disappeared when looking at the Low Des coef animals, that had high viremia levels and had a reduced growth, in comparison to the better High Des coef animals. There are only small differences between the unfavorable AA animals and the favorable AB animals, with the most apparent difference in enriched B cell and T cell differentiation GO terms in the AA animals. The top different hub genes in the networks between the most and least desirable phenotype (LvHg versus HvLg, respectively) or genotype (AB versus AA, respectively) did not differ greatly in their connection to immune genes.

### Do WGCNA and PCIT methods agree with one another?

As seen earlier, the red module from the WGCNA analysis, annotated as a highly immune gene rich module, was negatively correlated with weight gain and more highly expressed in the AA animals. This agrees with the PCIT results, where immune response GO terms were more enriched in the correlates of the Lg over the Hg group and, to a lower extent, the AA versus AB animals. However, it is not predominantly the genes in the red module that were more tightly correlated in the PCIT analyses. Combining results across these analyses, it is clear that the immune response pathways regulated at 4dpi play a substantial role when defining the pig’s long-term phenotypic response to a PRRSv infection, as affirmed by Boddicker et al. [9].

Furthermore, in Fig. 3 it can be seen that modules of co-expressed genes are correlated with weight gain, viral load or neither of these two, but never with weight gain and viral load simultaneously. A significant correlation of the Des coef with the ME in the magenta and lightcyan module was driven by only one of the two phenotypes. The PCIT results indicated that in this 4dpi-0dpi dataset it is more difficult to find co-expressed gene clusters correlated with both phenotypes in a desired direction. From the earliest PHGC trials it was clear that VL and WG were only poorly correlated; however, the SSC4 allele perfectly negatively correlated WG with VL [9, 10]. Other genomic influences would be predicted to influence just one not both traits. The greatest difference in GO term enrichment of the correlates of top 10 hub genes for both VL and WG are both immune response terms: they are more strongly connected with the desired phenotype for VL, but at the same time, also with the undesired phenotype for WG.

## Conclusion

In this study, it became clear that a gene-by-gene linear modeling analysis was not sufficiently sensitive to find expression differences in the blood early after infection between animals with a different weight gain or viral load after a PRRSv infection or between animals with the favorable and unfavorable WUR genotype. However, the network-level approaches WGCNA and PCIT successfully found co-expressed immune response genes at 4dpi when compared to 0dpi to be correlated as a group with weight gain, viral load or WUR genotype. Thus these genes can be useful targets in future efforts to select for disease-resistant pigs.

### Availability of supporting data

All microarray experimental data are MIAME compliant and have been deposited in Gene Expression Omnibus (GEO) with the accession number: GSE69515 (http://www.ncbi.nlm.nih.gov/geo/query/acc.cgi?acc=GSE69515).

## Additional files

Additional file 1: Table S1. List of DE genes (FDR ≤ 0.10) between 4dpi and 0dpi. The Oligo ID is a unique microarray probe name of the transcript. HGNC, extra annotation and HGNC extra annotation are different annotations. For all genes, the log_2_ fold change is shown, as well as the nominal *p*-value and q-value for that fold change. Additional file 2: Table S2. List of DE genes (FDR ≤ 0.10) between 7dpi and 0dpi. The Oligo ID is a unique microarray probe name of the transcript. HGNC, extra annotation and HGNC extra annotation are different annotations. For all genes, the log_2_ fold change is shown, as well as the nominal *p*-value and q-value for that fold change. Additional file 3: Table S3. List of genes selected for validation of the microarray results with RNAseq data. 31 and 15 genes were differentially expressed in the micro-array and found in the RNAseq experiment, and these were used to validate the DE between 4dpi and 0dpi and between 7dpi and 0dpi, respectively. For every gene, the probe is indicated by the unique Oligo ID, and the HGNC of each gene is shown. For both the microarray and RNAseq data, the DE is specified by the log_2_ fold change on the respective day, as well as the nominal *p*-value and q-value for that fold change. The last three columns show the comparison between the two datasets: when the gene fold change in both datasets goes in the same direction, it is indicated with a “+”, if this change in the RNAseq is significant, it is indicated with a “+”, and if both direction and significance are similar for both datasets, the validation is confirmed and indicated with a “+”. Additional file 4: Figure S1. Modules and their correlations with weight and blood viremia on specific days. Weight was measured at 0, 7, 14, 19/21, 28, 35 and 40/42 dpi, viremia was examined at 4, 7, 11, 14 and 19/21 dpi. Additional file 5: Figure S2. Stability analysis for the 4dpi-0dpi dataset. From the 4dpi-0dpi dataset 50 random samplings were performed and modules were created to evaluate the stability of the original full dataset. Additional file 6: Figure S3. WGCNA clusters shown with BioLayout Express 3D. Nodes are colored according to the module color assigned by WGCNA. All transcripts with a Pearson correlation of R^2^ > 0.70 are kept. Additional file 7: Table S4. Annotation of significant WGCNA modules in the 4dpi-0dpi comparison using DAVID. GO term descriptions are shown for those modules in the 4dpi-0dpi dataset that were significantly correlated (*p*-value < 0.10) with at least one of the phenotypes examined. Total number of transcripts in a module is given, as well as how many of them could be linked to a human Ensembl Gene ID and of those, how many were recognized by DAVID. An enrichment score (Enrichment score *p*-value ≥ 1.30) for each annotation is given as well. In orange is the 4dpi-0dpi red module, discussed in more detail in the discussion. Additional file 8: Table S5. PCIT results for the 4dpi-0dpi comparison for the four different contrasts. Contrasts made were done according to genotype, WG, VL and Des coef. The top 10 hub genes for every comparison are shown. The Oligo ID column shows a unique microarray probe name of the transcript. The HGNC column shows the annotated gene name, when available. The PCIT score represents the difference in amount of correlates. The group with the most correlates is named in the first column. Module color is the color for the hub gene given by WGCNA in the 4dpi-0dpi dataset. Additional file 9: Table S6. Pigoligoarray probes present in the red module of the 4dpi-0dpi dataset. The Oligo ID column shows a unique microarray probe name of the transcript. HGNC, extra annotation and HGNC extra annotation are different annotations. The Gene Significance (GS) and its *p*-value for each gene in relation to the trait WG, VL and WUR genotype in the depicted module is shown. The higher the absolute value of this GS, the more biologically significant the gene is for the module with regard to the trait. In orange are those genes discussed in more detail.

## Abbreviations

BE3D: BioLayout Express 3D
CTEN: Cell type enrichment
DAVID: Database for Annotation, Visualization and Integrated Discovery
DE: Differential expression
Des coef: Desirability Coefficient
DH: Differential hubbing
dpi: Days post infection
FDR: False Discovery Rate
GEO: Gene Expression Omnibus
GO: Gene Ontology
H: High responders
HvHg: High VL with maximal WG.
HvLg: High VL with reduced WG
IACUC: Institutional Animal Care and Use Committee
L: Low responders
LIMMA: Linear Models for Microarray Data
LvHg: Low VL with maximal WG
LvLg: Low VL with reduced WG
ME: Module eigengene
NCBI: National Center for Biotechnology Information
NHS: N-hydroxysuccinate
PCIT: Partial Correlation and Information Theory
PHGC: PRRS Host Genetics Consortium
PRRSv: Porcine Reproductive and Respiratory Syndrome virus
QTL: Quantitative Trait Locus
SD: standard deviation
SNP: Single Nucleotide Polymorphism
SSC4: *Sus* scrofa chromosome 4
TCID50: 50 % Tissue Culture Infective Dose
TMM: Trimmed Mean of M-values
VL: Viral load
WG: Weight gain
WGCNA: Weighted Gene Co-expression Network Analysis
WUR: WUR10000125
