# Supplementary figures and images for: Whole blood microarray analysis of pigs showing extreme phenotypes after a porcine reproductive and respiratory syndrome virus infection
Source: BMC Genomics. 2015 Jul 10;16(1):516. doi: 10.1186/s12864-015-1741-8 (PMC4496889; doi:10.1186/s12864-015-1741-8)

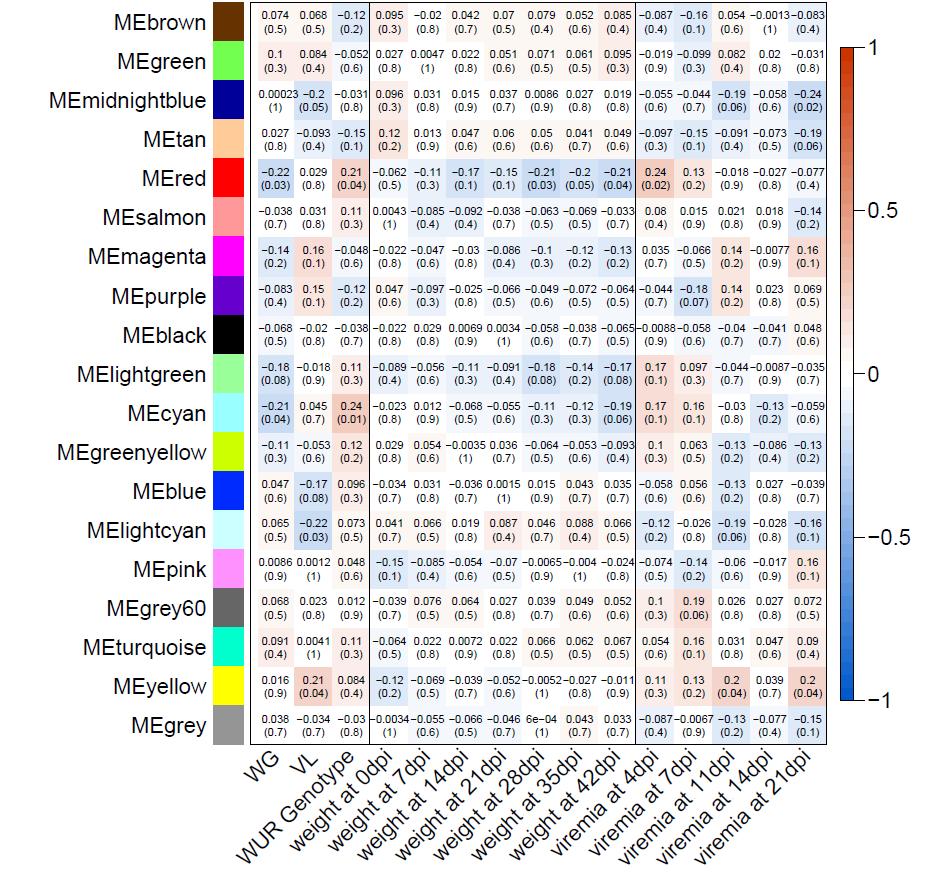

Supplement: Additional file 4: Figure S1. — Modules and their correlations with weight and blood viremia on specific days. Weight was measured at 0, 7, 14, 19/21, 28, 35 and 40/42 dpi, viremia was examined at 4, 7, 11, 14 and 19/21 dpi. [file 12864_2015_1741_MOESM4_ESM.jpeg]

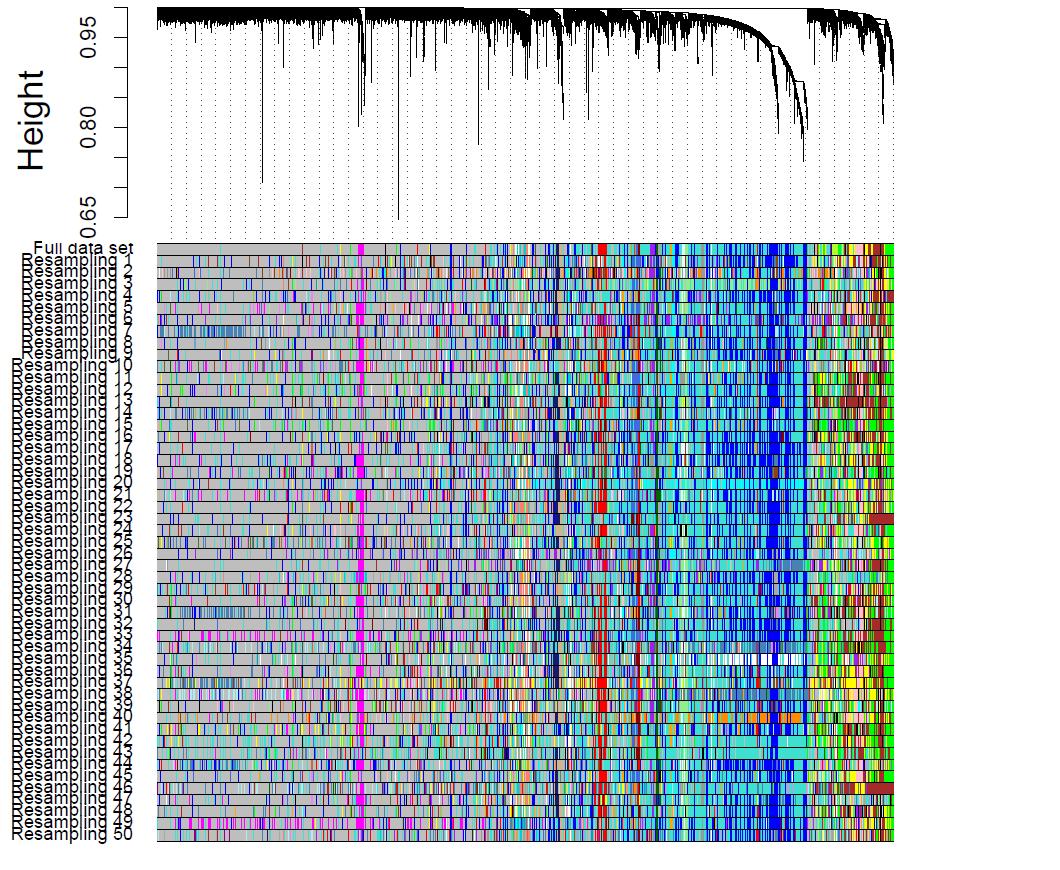

Supplement: Additional file 5: Figure S2. — Stability analysis for the 4dpi-0dpi dataset. From the 4dpi-0dpi dataset 50 random samplings were performed and modules were created to evaluate the stability of the original full dataset. [file 12864_2015_1741_MOESM5_ESM.jpeg]

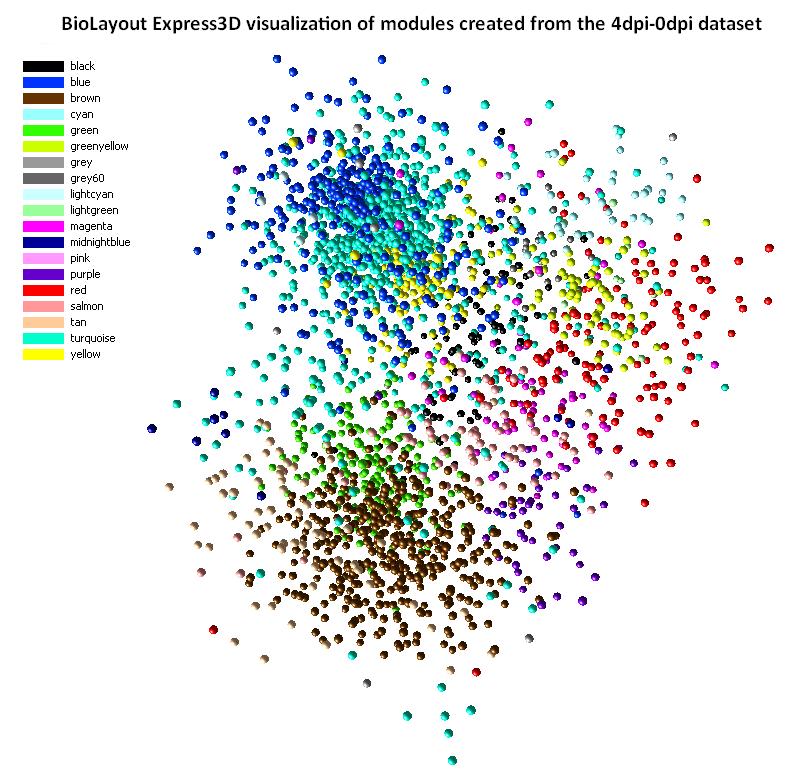

Supplement: Additional file 6: Figure S3. — WGCNA clusters shown with BioLayout Express 3D. Nodes are colored according to the module color assigned by WGCNA. All transcripts with a Pearson correlation of R2 > 0.70 are kept. [file 12864_2015_1741_MOESM6_ESM.jpeg]
